# NF-E2-Related Factor 2 (Nrf2) Ameliorates Radiation-Induced Skin Injury

**DOI:** 10.3389/fonc.2021.680058

**Published:** 2021-08-23

**Authors:** Jiao Xue, Chenxiao Yu, Yiting Tang, Wei Mo, Zhicheng Tang, Wenjiong Sheng, Yang Jiao, Wei Zhu, Jianping Cao

**Affiliations:** ^1^State Key Laboratory of Radiation Medicine and Protection, Soochow University, Suzhou, China; ^2^Department of Radiation Oncology, The First Affiliated Hospital of Soochow University, Suzhou, China; ^3^School of Radiation Medicine and Protection, Medical College of Soochow University, Suzhou, China; ^4^Department of Radiotherapy and Oncology, The Second Affiliated Hospital of Soochow University, Suzhou, China; ^5^Institute of Radiation Oncology, Soochow University, Suzhou, China

**Keywords:** radiation-induced skin injury (RISI), NF-E2-related factor 2 (Nrf2), reactive oxygen species (ROS), radiotherapy, antioxidant response

## Abstract

Radiation-induced skin injury (RISI) commonly occur in cancer patients who received radiotherapy and is one of the first clinical symptoms after suffering from nuclear exposure. Oxidative damage is the major causes of RISI. Nuclear factor erythroid 2-related factor 2 (Nrf2) is considered as a key mediator of the cellular antioxidant response. However, whether Nrf2 can alleviate RISI after high-dose irradiation remains unknown. In this study, we demonstrated that Nrf2-deficient (*Nrf2* ^-/-^) mice were susceptible to high-dose irradiation and adenovirus-mediated overexpression of Nrf2 (ad-Nrf2) protected against radiation in skin cells. Overexpression of Nrf2 attenuated the severity of skin injury after high-dose electron beam irradiation. To uncover the mechanisms of Nrf2 involved in RISI, mRNA sequencing technology was performed to analyze the mRNA expression profiles of Ad-Nrf2 skin cells following radiation. The results revealed that a total of 127 genes were significantly changed, 55 genes were upregulated, and 72 genes were downregulated after Nrf2 overexpression. GSEA showed that Nrf2 was associated with positive regulation of genes involved in the reactive oxygen species pathway after radiation. Taken together, this study illustrated the role of Nrf2 in RISI and provided potentially strategies for ameliorating RISI.

## Introduction

Radiation is widely used in industry, medicine and science and may significantly increase uncontrolled exposure to radiation (1, 2). Since the skin is the first tissue through which external radiation particles enter the human body, it is vulnerable to radiation-induced injury. Moreover, radiotherapy is applied to over 70% of cancer patients, either alone or in combination with other treatments (3, 4). In fact, radiation-induced dermatitis remains a serious concern that may limit the duration and dose of radiotherapy (3, 5). Radiation can both directly induce DNA double strand breaks (DSBs) and indirectly produce reactive oxygen species (ROS) and reactive nitrogen species (RNS), including hydroxyl radicals, superoxide anions, hydrogen peroxide and nitrogen dioxide (6, 7). These reactive molecules undergo interconversion, induce DNA damage and eventually lead to acute and/or chronic skin injuries (7, 8). Thus, elimination of different kinds of oxidative species is a vital way to protect skin from radiation and prevent RISI.

When exposed to radiation, human cells launch a complex antioxidant response involving multiple antioxidant enzymes, such as superoxide dismutases (SODs), catalase and glutathione peroxidase (GPx) (9). However, a single antioxidant enzyme is unable to eliminate all kinds of oxidative species (10). NF-E2-related factor 2 (Nrf2), a member of the NF-E2 family of basic leucine zipper transcription factors, is considered a key mediator in regulating the antioxidant response (11). Nrf2 heterodimerizes with members of the sMaf protein family, binds to the antioxidant response element (ARE) in the promoter region of multiple antioxidant and/or detoxification enzymes, and activates the transcription of these genes (12). Previous studies have suggested that the antioxidant capacity of Nrf2 is mediated through ROS-eliminating enzymes, and Nrf2 is thought to protect against stress-induced cell death (13). Pharmacological induction of Nrf2 before radiation exposure is thought to prevent radiation-induced dermatitis (14). The role of Nrf2 in high-dose radiation-induced skin injury remains unknown, and there is no strong evidence that exogenous supplementation with Nrf2 is capable of attenuating RISI.

In this study, we found that skin tissues and primary skin cells from Nrf2-deficient (*Nrf2* ^-/-^) mice were more susceptible to 20 Gy irradiation than those from WT (*Nrf2* ^+/+^) mice. Adenovirus-mediated overexpression of Nrf2 reduced ROS and conferred protection against high-dose irradiation in skin cells and rat skin tissues. To understand the distinct mechanisms involved in radiation-induced skin injury, the mRNA expression profiles of Nrf2-overexpressing skin cells and control skin cells were analyzed using mRNA sequencing technology. In conclusion, we illustrated the key role of Nrf2 in protecting against high-dose radiation-induced skin injury and the underlying mechanisms. This study may provide new strategies to ameliorate skin injury for people in nuclear accidents and patients receiving radiotherapy.

## Methods

### Reagents and Adenovirus

Bovine serum albumin, Hoechst 33342 and DAPI were purchased from Beyotime (Shanghai, China). The Nrf2-overexpression adenovirus was designed and constructed by GeneChem (Shanghai,China).

### Animal Studies

Protocols for experiments involving animals were approved by the Animal Experimentation Ethics Committee at Soochow University (China). The C57B L/6 Nrf2 knockout (*Nrf2^-^*/-) mice were a kind gift from Dr. Peng Cao (Jiangsu Research Institute of Traditional Chinese Medicine, Nanjing, China). The mice were originally obtained from The Jackson Laboratory (stock number: 017009). Nrf2-/- mice were detected by PCR (**Figure S1A**) with genotyping protocol presented on the website of the Jackson Laboratory (https://www.jax.org/Protocol?stockNumber=017009&protocolID=26266).

Male Sprague Dawley (SD) rats (4 weeks of age) were purchased from the Shanghai SLAC Laboratory Animal Co., Ltd. These animals were housed in a pathogen-free environment at the facilities of Medical School of Soochow University. Animals were anesthetized with an intraperitoneal injection of ketamine (75 mg/kg) and xylazine (10 mg/kg) (Sigma), and the hair on the gluteal region of the rats was shaved using a razor. Animals were immobilized with adhesive tape on a plastic plate to minimize motion during radiation exposure. A 3-cm-thick piece of lead was used to shield the animals and localize the radiation field (3×4 cm). Irradiation was administered to the treatment area at a dose rate of 750 cGy/min using a 6-MeV electron beam accelerator (Clinac 2100EX, Varian Medical Systems) as we reported previously (15–22).

SD rats randomly received one of the following treatments (n = 5): (1) a 200-μl subcutaneous injection of phosphate-buffered saline (PBS); (2) a subcutaneous injection with 200 μl of control adenovirus (1 × 10^10^ PFU/ml); (3) a subcutaneous injection with 200 μl of Nrf2 overexpression adenovirus (1 × 10^10^ PFU/ml). Animals were randomly selected with respect to control littermates. No animals were excluded from analysis. Skin reactions were graded at regular intervals from 1 (no damage) to 5 (severe damage) using the semiquantitative skin injury scale as previously described (15–22).

### Human Tissues

Human skin samples were obtained from a victim of a radiation accident involving an iridium gammagraphy radioactivity source. The patient had been irradiated with ~300 Gy at the center of the skin surface. The skin samples were obtained ~180 days after radiation exposure. Informed consent for sample collection was obtained from the patient. And this study was approved by the Ethics Committee at Soochow University.

### Cell Culture and Irradiation

Human keratinocyte HaCaT and human skin fibroblast WS1 cells were kind gifts from Prof Hongying Yang (Soochow University). The cells were maintained in Dulbecco’s Modified Eagle’s Medium (DMEM). Primary skin cells of mice were isolated from skin of adult mice (5-6 weeks of age) using the procedures as reported (22, 23). Primary skin cells were maintained in DMEM. All culture media were supplemented with 10% FBS (Gibco). Cells were grown at 37°C in 5% CO2 incubators. Cells were exposed to different dosages (5 or 20 Gy) of ionizing radiation using X-ray linear accelerator (RadSource) at a fixed dose rate of 1.15 Gy/min.

### Mitochondrial Membrane Potential Assay

Mitochondrial membrane potential was measured using JC-1 staining (Cayman). Cells were pre-infected with indicated adenovirus before receiving 20 Gy of X-ray irradiation. 24 h after irradiation, the cells were incubated for 30 min in the dark with JC-1 dissolved in serum-free medium at 37°C. Nuclei were counterstained with DAPI.

### ROS Generation Assay

ROS levels were determined using the ROS-sensitive dye 2, 7-dichlorofluoresceindiacetate (DCF-DA) (Nanjing Jiancheng Bioengineering Institute). HaCaT and WS1 cells were washed with PBS and incubated with DCF-DA (10 μM) for 30 min. The level of DCF fluorescence, reflecting the concentration of ROS, was measured by a fluorescence microscope. For skin tissues, the level of DCF fluorescence was measured at 488 nm using a 96-well plate reader.

### Malondialdehyde (MDA) Concentration Measurement 

Tissue MDA levels were determined by thiobarbituric acid (TBA) reaction. The optical density (OD) was measured at a wavelength of 532 nm. The assay depended on the measurement of the pink color produced by the interaction of barbituric acid with MDA generated as a result of lipid peroxidation. The colored reaction with 1,1,3,3- tetraethoxy propane was used as the primary standard. Fresh skin samples were homogenized with 50 mM phosphate buffer (pH 7.4). Then, homogenates were centrifuged at 12,000 × g for 10 min at 4°C. MDA levels were expressed as a nano mol per milligram of protein (nmol/mg protein).

### Immunofluorescence Assay

Cells were washed with PBS, fixed with 4% formaldehyde and blocked with 1% BSA/PBS for 1 h at room temperature. Then, primary antibodies against Nrf2 (Abcam; ab62352) or γH2AX (Abcam; ab81299) were incubated with cells overnight at 4°C and secondary antibodies conjugated with Cy3 (Beyotime, Nantong, China; A0516) were employed for 1 h at room temperature. DAPI (Sigma) was used to stain the nuclei and images were captured by an UltraViewVoX confocal microscopy (PerkinElmer, Waltham, MA).

### Cell Death Assay

Cells were pre-infected with the adenovirus 24 h before receiving irradiation. Cell death was measured using the 7-AAD/Annexin-V double staining apoptosis kit (BD Biosciences) by flow cytometry (BD Biosciences). Each group was set up in triplicate.

### EdU Assay

Cell proliferation staining was performed using an EdU kit (BeyoClick EdU Cell Proliferation Kit with Alexa Fluor 488, Beyotime, China). Briefly, HaCaT or WS1 cells (2 × 104 cells/well) were seeded in 96-well plates and cultured, respectively. Subsequently, cells were incubated with EdU for 3 h, fixed with 4% paraformaldehyde for 15 min, and permeated with 0.3% Triton X-100 for another 15 min. The cells were incubated with the Click Reaction Mixture for 30 min at room temperature in a dark place and then incubated with Hoechst 33342 for 10 min.

### Cell Senescence Assay

The most widely used biomarker for senescent and aging cells is senescence-associated beta-galactosidase (SA-beta-gal), which is defined as beta-galactosidase activity detectable at pH 6.0 in senescent cells. Forty eight hours after treatment, cells were fixed in 2% fomaldehyde/0.2% glutaraldehyde for 5 min at room temperature. Cells were rinsed with PBS and β-galactosidase staining solution containing 20 mg/mL X-gal (Beyotime) was added. Cells were incubated for 6-10 h at 37°C incubator without CO_2_.

### Western Blot Assay

Cells were treated with adenovirus before irradiation with 20 Gy of radiation. The cells were then washed twice with ice-cold PBS and directly lysed in 200 µl of cell lysis buffer. The lysates were centrifuged at 12,000 × g and then loaded onto an SDS–PAGE gel. The samples were electrophoresed for 2 h and transferred onto PVDF membranes. After blocked with 5% non-fat milk in PBS-Tween 20 for 1 h at room temperature, the membranes were blotted with primary antibodies (Abcam: ab62352, ab76026). The membranes were then incubated with the appropriate HRP-coupled secondary antibody (Beyotime) at 1:2000 dilution for 1 h at room temperature. After the membranes were washed with TBST, the blots were incubated in ECL-plus (Beyotime, Nantong, China) and detected by FluorChem™ M System (Protein Simple, San Jose, CA).

### Hematoxylin and Eosin (H&E) Staining

Skin tissues were fixed in 10% neutral-buffered formalin and embedded in paraffin. Three-micrometer paraffin sections were deparaffinized and heat treated with citrate buffer (pH 6.0) for 7 min following an epitope retrieval protocol. The sections were stained with H&E (ZSGB-Bio, Beijing, China).

### Whole Transpriptome Sequencing

HaCaT cells were treated with Ad-NC or Ad-Nrf2 for 24 h and irradiated at a dose of 20 Gy. Equal quantities of cells from triplicates were mixed to generate one sample for each group. Total RNA was isolated from cells using Trizol (Sigma-Aldrich). Strand-specific libraries were generated using the Illumina TruSeq Stranded Total RNA Library Prep Kit with Ribo-Zero Gold. Paired-end 125-bp reads were generated on an Illumina HiSeq 2500 instrument at the Oebiotech.Co. Reads were aligned to the GRCh38.p7 genome using TopHat v2.1.1 with the library type option set to first strand. Fragments per kilobase of transcript per million mapped reads of known genes were calculated using eXpress v1.5.1. Raw data was uploaded to Sequence Read Archive (SRA) with bioproject accession PRJNA694347.

### Statistics

The data were evaluated using either unpaired two-sided Student’s t-tests or one-way analysis of variance to determine statistical significance after confirming that the data met appropriate assumptions (normality, homogeneous variance, and independent sampling). For all *in vitro* experiments, three biological replicates were analyzed. For all *in vivo* experiments, five biological replicates were analyzed for each condition. Statistical analysis was performed using Prism 6 software (GraphPad Software, La Jolla, CA). Data are expressed as means “ standard error of the mean and considered significant if P < 0.05 (*) and P < 0.01 (**). For the animal study, skin injury was scored in a “blinded” manner.

## Results

### Increased Susceptibility to Radiation-Induced Skin Injury in *Nrf2*-Deficient Mice

**Figure 1:**
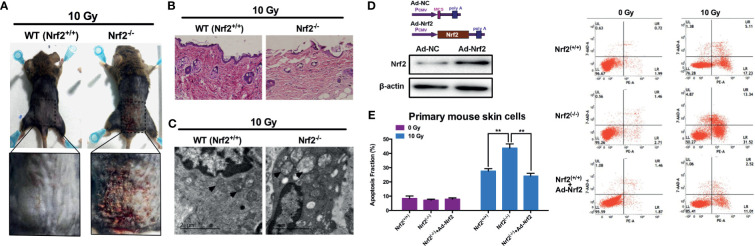
Increased susceptibility to radiation-induced skin injury in Nrf2-deficient mice. **(A)** Radiation-induced skin injury induced by 10 Gy electron beam radiation in WT (Nrf2^+/+^) and Nrf2-deficient (Nrf2^-/-^) mice. **(B)** HE staining of skin tissues in irradiated areas from WT (Nrf2^+/+^) and Nrf2-deficient (Nrf2^-/-^) mice. **(C)** Electron microscopy analysis of skin tissue from WT (Nrf2^+/+^) and Nrf2-deficient (Nrf2^-/-^) mice. Scale bar=2μm. **(D)** Nrf2 was overexpressed after Ad-Nrf2 treatment. **(E)** Cell death rate of primary skin cells from WT (Nrf2^+/+^) and Nrf2-deficient (Nrf2^-/-^) mice after Nrf2 overexpression and 10 Gy irradiation. ** means p < 0.01.

We first analyzed the effect of Nrf2 depletion on the radiosensitivity of mouse skin using Nrf2 wild-type (WT, *Nrf2* ^+/+^) and Nrf2-deficient (*Nrf2* ^-/-^) mice (**Supplementary Figure 1**). A single dose of 10 Gy of irradiation was delivered to the skin in the gluteal region of *Nrf2* WT and deficient mice. In *Nrf2^-/-^* mice, cutaneous damage was observed at 3 d after irradiation. Skin injury reached a maximum at 10 d after irradiation. In contrast, in WT *Nrf2* ^+/+^ mice, radiation-induced skin injury was not observed (**Figure 1A** and **Supplementary Figure 2**). H&E staining indicated a loss of the epidermis and a disintegrated structure in the skin tissue of *Nrf2* ^-/-^ mice after irradiation, whereas the skin tissue of WT mice did not show this feature (**Figure 1B**). Electron microscopy analysis further revealed obvious swelling of mitochondria in the skin of Nrf2-deficient mice after irradiation (black arrow; **Figure 1C**).

To further evaluate the role of Nrf2 in radiation-induced cell death, primary skin cells from WT and *Nrf2* ^-/-^ mice were isolated and cultured. An Nrf2-overexpressing adenovirus was constructed and introduced into cultured primary skin cells, leading to an increase in the protein level of Nrf2 (**Figure 1D**). Forty-eight hours after 10 Gy X-ray irradiation, the cell death rate of skin cells from *Nrf2* ^-/-^ mice was significantly higher than that of WT skin cells (**Figure 1E**). Nrf2 reintroduction using adenovirus in skin cells from *Nrf2* ^-/-^ mice dramatically decreased the cell death rate (**Figure 1E**). These data provide important insights into the critical role of Nrf2 in preventing radiation-induced skin injury.

### Nrf2 Confers Protection Against Radiation in Skin Cells

**Figure 2:**
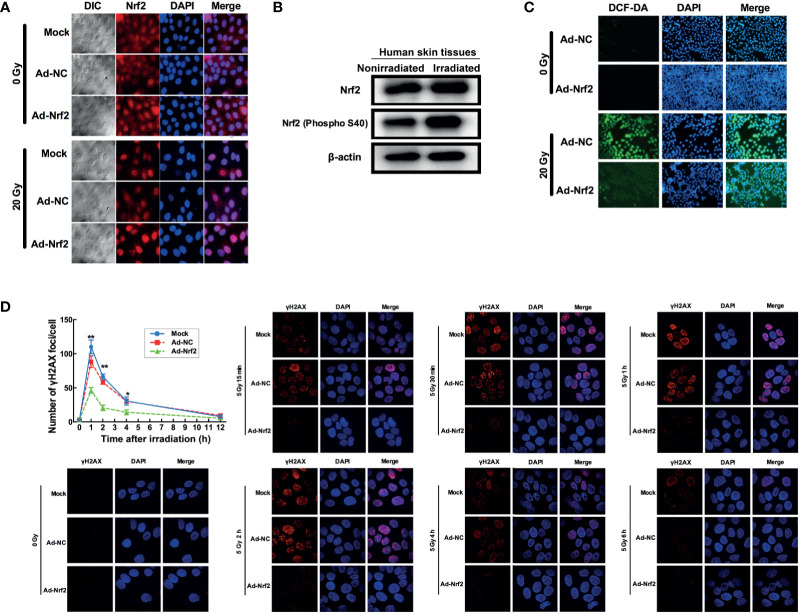
Nrf2 confers protection against radiation in skin cells. **(A)** Immunofluorescence assay detecting Nrf2 translocation after irradiation and Nrf2 overexpression. **(B)** Western blot analysis of Nrf2 and phosphorylated Nrf2 (S40) after irradiation. **(C)** ROS levels in skin cells after IR and Nrf2 overexpression were detected using an ROS-sensitive DCF-DA probe. **(D)** The dynamic repair process of DNA DSBs was measured by detecting nuclear gH2AX foci at several time points after 5 Gy of X-ray irradiation. * means p < 0.05, ** means p < 0.01.

We next explored the involvement of Nrf2 in the response to ionizing radiation. Human keratinocyte HaCaT cells were exposed to a single dose of 20 Gy of irradiation and then subjected to immunofluorescence detection of Nrf2. The results revealed that radiation induced a marked translocation of Nrf2 into the nucleus (**Figure 2A**), indicative of its involvement in the cellular response to radiation. Western blot analysis of irradiated and nonirradiated human skin tissues showed no change in the total level of Nrf2. Nevertheless, irradiated skin tissues expressed a higher level of phosphorylated Nrf2 than nonirradiated skin tissues (**Figure 2B**). These results indicated that irradiation modulates Nrf2 activity in human skin.

Because radiation generates free radicals, including ROS (17), we investigated whether free radicals are modulated by Nrf2 alteration after irradiation. Human keratinocyte HaCaT cells and human skin fibroblast WS1 cells preinfected with Nrf2 adenovirus showed a marked reduction in radiation-induced elevation in ROS levels (**Figure 2C** and **Supplementary Figure 3**). Since free radicals also exert DNA damage, we further explored whether Nrf2 influences the dynamic repair process of DNA double-strand breaks (DSBs) induced by radiation in HaCaT cells. At 1, 2 and 4 hours after irradiation, the number of foci in the Nrf2 adenovirus-infected groups dropped to 57.1% (*P*< 0.01), 45% (*P* < 0.01) and 41.67% (*P* < 0.01) of the number in the control adenovirus-infected group, respectively (**Figure 2D**). These results demonstrated that overexpression of Nrf2 induced ROS elimination after irradiation and attenuated radiation-induced DNA damage in human skin cells.

### Nrf2 Reduces Cell Death and Senescence After Irradiation

**Figure 3:**
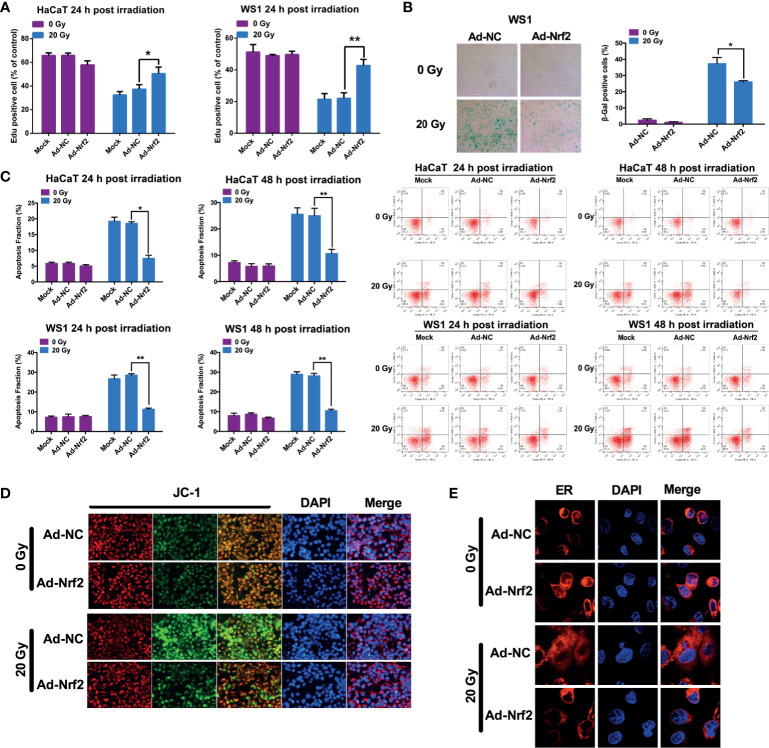
Nrf2 restores cell viability and reduces cell death and senescence after irradiation. **(A)** The proliferation of HaCaT and WS1 cells was measured in an EdU incorporation assay at 24 hours after irradiation. **(B)** The cell death rate of HaCaT and WS1 cells was detected using Annexin-V/7-AAD staining. **(C)** The senescence of WS1 cells was evaluated using β-galactosidase staining. **(D)** The mitochondrial membrane potential in HaCaT cells was evaluated using a JC-1 staining assay. **(E)** The ER structure was visualized using ER-Tracker Red. * means p < 0.05, ** means p < 0.01.

To investigate the effect of Nrf2 on the viability of skin cells, cells were infected with Nrf2 adenovirus or control adenovirus prior to radiation exposure. HaCaT and WS1 cells infected with Nrf2 adenovirus exhibited significantly higher viability than cells infected with control adenovirus following radiation (**Figure 3A** and **Supplementary Figure 4**). We next investigated whether overexpression of Nrf2 was associated with a decreased cell death rate in skin cells. As shown in **Figure 3B**, forced expression of Nrf2 did not affect the cell death rate of HaCaT and WS1 cells that were not exposed to irradiation. In contrast, Nrf2 overexpression significantly decreased the cell death rate of HaCaT cells that were exposed to 20 Gy irradiation. In radiation-sensitive WS1 cells, Nrf2 overexpression exhibited a pronounced protective capacity by reducing radiation-induced cell death by up to 52% (**Figure 3B** and **Supplementary Figures 6A, B**). These results demonstrated that Nrf2 reduces the cell death of skin cells caused by irradiation. To explore whether Nrf2 plays a role in radiation-induced cell senescence, β-galactosidase staining was performed. Irradiation caused a significant increase in β-galactosidase staining in WS1 cells, and Nrf2 overexpression significantly decreased cell senescence (**Figure 3C**). These results indicated that Nrf2 reduces the irradiation-induced senescence of skin cells.

Mitochondrial functional failure, including the loss of mitochondrial integrity and changes in mitochondrial membrane potential, is one of the most important factors causing cell death. Nonirradiated HaCaT cells predominantly showed red fluorescence by JC-1 staining, whereas a substantial proportion of cells shifted to green fluorescence after irradiation, indicating that the mitochondrial membrane potential was reduced. HaCaT cells with forced expression of Nrf2 exhibited less of a shift from red to green fluorescence, indicating that the mitochondrial membrane potential can be maintained by Nrf2 overexpression after ionizing radiation (**Figure 3D**). These results demonstrated that Nrf2 protects mitochondria against ionizing radiation. Next, we explored the protective effect of Nrf2 on the endoplasmic reticulum (ER) structure using ER-Tracker Red. Nonirradiated HaCaT cells showed clear paranuclear staining of the ER, with a clear ER structure, which was attenuated and morphologically changed after 20 Gy of irradiation (**Figure 3E**). However, Nrf2 adenovirus-infected cells showed a paranuclear ER-Tracker Red distribution, which was morphologically similar to that of nonirradiated cells, indicating that the integrity of the endoplasmic reticulum can be maintained by Nrf2 overexpression after ionizing radiation (**Figure 3E**).

### Nrf2 Overexpression Ameliorates Radiation-Induced Skin Injury in Rat Models

**Figure 4:**
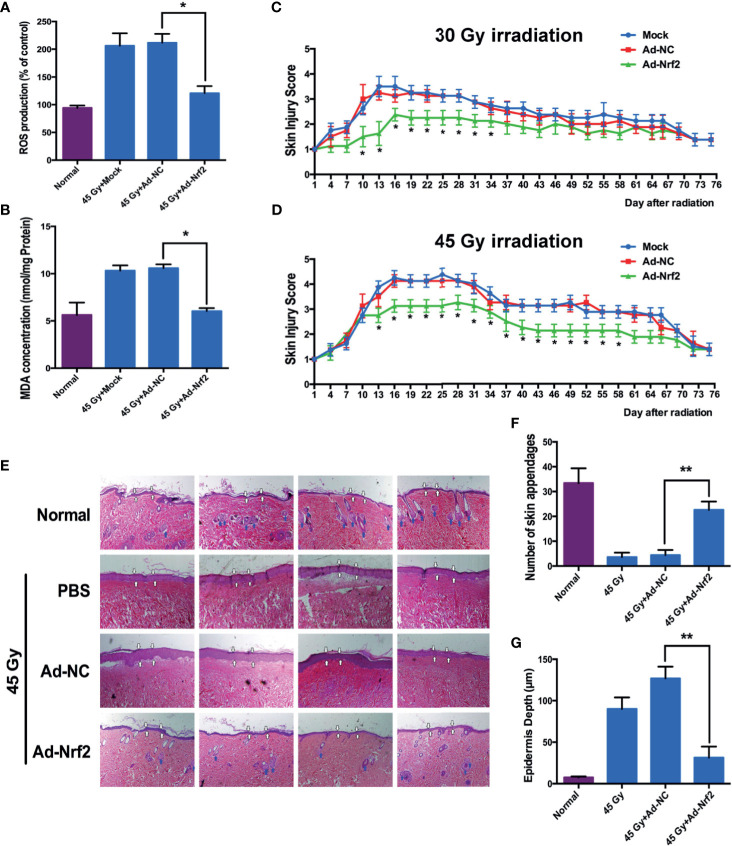
Nrf2 overexpression ameliorates radiation-induced skin injury in rat models. Rat gluteal skin was irradiated with an electron beam followed by subcutaneous injection of control adenovirus, Nrf2 adenovirus or PBS (six animals per group). **(A)** ROS levels in rat skin tissues from the irradiated area were detected 3 days after IR. **(B)** Lipid peroxidation of rat skin tissues from the irradiated area was detected using MDA 3 days after IR. **(C, D)** Skin injury was measured using a semiquantitative score of 1 (no damage) to 5 (severe damage). **(E)** Representative hematoxylin and eosin (HE) staining of rat skin at 75 days after irradiation. **(F)** Number of skin appendages in each group. **(G)** Epidermal thickness in each group. * means p < 0.05, ** means p < 0.01.

Irradiation (45 Gy and 30 Gy) was delivered to the gluteal region of rats to establish rat RISI models. Irradiation at 45 Gy significantly increased skin ROS levels at 3 d after treatment, whereas infection with Nrf2, but not the control adenovirus, significantly reduced the generation of ROS (**Figure 4A**). Because radiation-induced ROS results in oxidative damage to lipids, aggravating the progress of skin injury, we measured the concentration of MDA in skin tissues after 45 Gy of irradiation to test whether the overexpression of Nrf2 affects radiation-induced lipid peroxidation. As shown in **Figure 4B**, infection with Nrf2 adenovirus resulted in an approximately 1.8-fold significant decrease in MDA levels compared with the control adenovirus. These results indicated that Nrf2 overexpression attenuates ROS generation and lipid peroxidation *in vivo*. Two radiation-induced skin injury models (30 and 45 Gy electron beam irradiation of rat skin) were applied to evaluate the role of Nrf2 in wound healing. After irradiation, injuries to skin tissues were graded on a scale of 1 (no damage) to 5 (severe damage) as described previously (16, 17). After exposure to 30 Gy of irradiation, cutaneous damage to rat skin began at 15 d after irradiation, although the damage was less severe than that after exposure to 45 Gy of irradiation. Adenovirus-mediated Nrf2 overexpression in rats ameliorated radiation-induced skin injury compared with control adenovirus- and PBS-treated rats (**Figure 4C**). In rats exposed to 45 Gy of irradiation, radiation-induced skin injury was significantly less severe at 13 d after irradiation in the Nrf2-overexpressing group than in the PBS-treated group (**Figure 4D** and **Supplementary Figure 5**). Although infection with Nrf2 adenovirus showed a similar skin wound as PBS-treated or control adenovirus-infected rats at 75 d postirradiation, Nrf2 overexpression attenuated epidermal hyperplasia (white arrow) and maintained skin appendages (blue arrow), which were often destroyed by irradiation (**Figures 4E–G**).

### Nrf2 Changes the Gene Expression Profile in Skin Cells After IR

**Figure 5:**
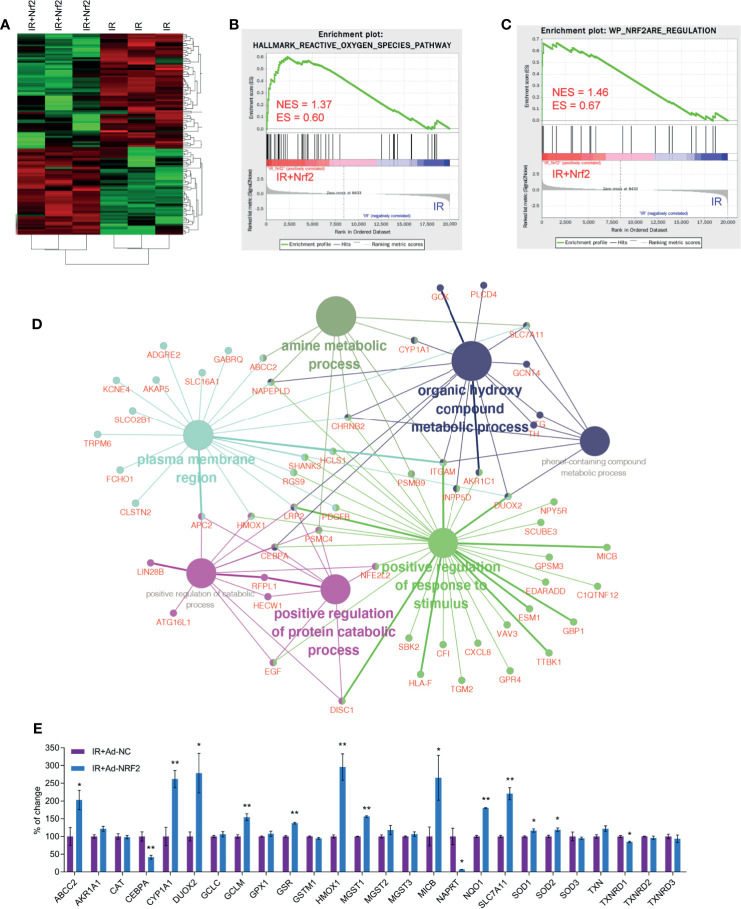
Nrf2 changes the gene expression profile in skin cells after IR. Transcriptome sequencing was performed for HaCaT cells overexpressing Nrf2. **(A)** Heatmap of cluster analysis based on sequencing results. **(B)** GSEA plot showing normalized enrichment scores (NESs) for reactive oxygen species pathway signatures using RNA-seq data from IR+Nrf2- and IR-treated skin cells. **(C)** GSEA plot showing normalized enrichment scores (NESs) for Nrf2 ARE regulation signatures using RNA-seq data from IR+Nrf2- and IR-treated skin cells. **(D)** GO enrichment analysis of differentially expressed upregulated and downregulated genes based on RNA-seq data with adjusted P-values. **(E)** Real-time PCR analysis of the mRNA levels of Nrf2 downstream genes after IR+Nrf2 and IR treatment. * means p < 0.05, ** means p < 0.01.

Whole transcriptome sequencing technology was used to clarify the mechanism of RISI mitigation mediated by Nrf2. HaCaT cells overexpressing Nrf2 and control HaCaT cells were exposed to 20 Gy of irradiation, RNA was extracted, and gene profiling was performed. Statistical analysis indicated that the expression of a total of 127 genes were changed significantly after Nrf2 overexpression, 55 of which were upregulated, and 72 of which were downregulated. We used a heatmap to show these differences (**Figure 5A**). The 30 genes with the most significant upregulation and the 30 genes with the most significant downregulation are listed in **Tables 1**, **2**, respectively. The GO database explains the role of eukaryotic genes and proteins in cells by creating a set of control words with dynamic forms, including three independent ontologies: biological process (BP), molecular function (MF), and cellular component (CC). The enriched gene sets of significantly regulation are shown in **Figure 5D**. GO analysis indicated that genes were closely related to biological processes involved in the organic hydroxy compound metabolic process, positive regulation of response to stimulus, positive regulation of protein catabolic process, amine metabolic process, positive regulation of protein catabolic process (**Figure 5D** and **Figure S5**). These results show that the effect of RISI mitigation by Nrf2 involves multiple signaling pathways. Moreover, GSEA showed that IR+Nrf2 was associated with positive regulation of genes involved in the reactive oxygen species pathway and Nrf2/ARE pathway (**Figures 5B, C**). As gene sets that respond to oxidative stress were enriched, Nrf2 downstream gene expression levels were further examined. The expression levels of ABCC2, CYP1A1, DUOX2, GCLM, GSR, HMOX1, MGST1, MICB, NQO1, SLC7A11, SOD1 and SOD2 were significantly upregulated. However, the expression of some of the Nrf2 downstream targets, including GPX1 and catalase (CAT), did not change significantly. Surprisingly, the expression of CEBPA, NAPRT and TXNRD1 was downregulated dramatically after IR and Ad-Nrf2 compared with IR alone (**Figure 5E**).

**Table 1:** Downregulated genes between Ad-NC+20 Gy radiation and Ad-Nrf2+20 Gy radiation.

GENNE_ID	Fold change	GENE_NAME	UP_dOWN
5988	17.80	RFPL1	Up
200523	16.87	TEX37	Up
8787	16.83	RGS9	Up
285973	16.01	ATG9B	Up
6566	14.49	SLC16A1	Up
10316	14.13	NMUR1	Up
3290	14.04	HSD11B1	Up
5309	14.00	PITX3	Up
51596	12.04	CUTA	Up
3684	9.37	ITGAM	Up
1645	8.66	AKR1C1	Up
284656	8.43	EPHA10	Up
3576	8.37	CXCL8	Up
2091	7.76	FBL	Up
1388	6.41	ATF6B	Up
375298	6.40	CERKL	Up
266743	5.87	NPAS4	Up
23704	3.98	KCNE4	Up
23072	3.35	HECW1	Up

**Table 2:** Downregulated genes between Ad-NC+20 Gy radiation and Ad-Nrf2+20 Gy radiation.

GENE_ID	Fold change	GENE_NAME	Up_Down
5549	O.50	PRELP	Down
8908	0.50	GYG2	Down
58538	0.49	MPP4	Down
3892	0.49	KRT86	Down
445577	0.49	C9orf129	Down
7052	0.49	TGM2	Down
731220	0.48	RFX8	Down
10231	0.48	RCAN2	Down
94274	0.48	PPP1R14A	Down
117248	0.47	GALNT15	Down
102725009	0.47	LOC102725009	Down
54102	0.46	CLIC6	Down
2166	0.46	FAAH	Down
5155	0.46	PDGFB	Down
127707	0.46	KLHDC7A	Down
11082	0.45	ESM1	Down
10297	0.44	APC2	Down
10815	0.44	CPLX1	Down
285148	0.44	IAH1	Down

## Discussion

Radiation-induced skin injury (RISI) is one of the most common side effects of radiotherapy for cancer, affecting approximately 95% of patients receiving radiotherapy, especially in the management of head and neck cancers and breast cancers (3). Additionally, RISI is among the first symptoms to occur after radiation exposure during nuclear accidents. Skin effects can be divided into acute reactions, which occur within days of initiating exposure, and late effects, which often become apparent months to years after irradiation (23, 24). The involvement of an antioxidative response to radiation has been extensively reported, and the use of antioxidants could mitigate radiation-induced skin injury (25–29). Papers from our group have shown that superoxide dismutases (SODs) and peroxiredoxin 6 (PRDX6) reduced ROS levels and contributed to the amelioration of radiation-induced skin injury (15, 16). However, ionizing radiation induces several types of free radicals that may undergo instantaneous interconversion (17, 24, 30, 31). Although the simultaneous elimination of all kinds of free radicals is difficult, it will be an appropriate strategy to protect against or prevent RISI. Thus, finding a molecular switch that can eliminate different forms of free radicals may bring hope to address RISI. Nrf2 is considered one of the most important orchestrators of the cellular antioxidant response (32), which makes it an ideal target to treat RISI. However, the role of Nrf2 in high-dose radiation-induced skin injury is not fully understood. In the current study, we demonstrated that Nrf2-/- mouse skin exhibits susceptibility to irradiation. Forced reintroduction of Nrf2 in primary skin cells from Nrf2-/- mice increased cell viability and protected them from cell death induced by irradiation. Posttranslational modifications of Nrf2, including phosphorylation (33, 34) and acetylation (35, 36), were reported to alter its transcriptional activity. After exposure to radiation, Nrf2 was activated by S40 phosphorylation and translocated to the nucleus. However, the protein kinase mediating S40 phosphorylation remains unclear and needs further study. Taken together, these results, along with those of other studies (14, 37), clearly demonstrate that Nrf2 plays a pivotal role during the process of RISI.

Pharmacological induction or activation of Nrf2 has been widely studied to prevent or protect against radiation-induced dermatitis in preclinical research both *in vitro* and *in vivo* (14, 37). Overexpression is a more effective way to increase Nrf2 in cells. Radiation-induced oxidative stress also conveys signals to the cell nucleus to induce stress responses such as cell growth arrest, senescence and cell death. Exogenous overexpression of Nrf2 by adenovirus eliminated radiation-induced ROS, accelerated DNA damage repair, and protected vital cellular organelles, including mitochondria and ER, from radiation. Furthermore, overexpression of Nrf2 in both human keratinocyte HaCaT cells and human skin fibroblast WS1 cells restored cell viability, eased cell senescence and decreased cell death induced by a single dose of 20 Gy X-ray radiation.

Radiation-induced ROS are a complex mixture including •OH, O_2_•-, •O_2_H, ONOO-, and H_2_O_2_. These radicals, together with secondary radicals derived in the superoxide-generating environment, are likely to cause oxidative damage to lipids, DNA and proteins (38). Skin is rich in lipids, proteins and DNA, which makes it one of the most sensitive and targeted organs for oxidative stress (39). Nrf2 downstream targets, including SOD1, SOD2, catalase (CAT), and glutathione peroxidases (GPXs), contribute to cellular defense against irradiation (32). Subcutaneous injection of Ad-Nrf2 significantly decreased ROS levels and MDA levels in rat skin 3 days after radiation exposure, which indicated that Nrf2 overexpression attenuated lipid peroxidation and ROS amplification. The severity of cutaneous damage was quantified by the RISI score (1-5) as described previously (15–17). Acute skin damage began 15 d after irradiation. Subcutaneous injection of Nrf2 adenovirus ameliorated acute skin injury compared with the control adenovirus- and PBS-treated groups. Skin fibrosis, including hyperpigmentation and hypopigmentation, is one of the major forms of late skin injury induced by radiation. The TGF-β signaling pathway is reported to be involved in radiation-induced fibrosis. However, the mechanism of radiation-induced fibrosis is not fully understood, and there are no efficient treatments. Nrf2 overexpression attenuated epidermal hyperplasia and maintained skin appendages, showing that skin fibrosis was also reduced.

Whole transcriptome sequencing technology was used to uncover the underlying mechanism of Nrf2 in RISI. GO enrichment analysis showed that the response to oxidative stress was upregulated, as expected. Several well-defined target genes of Nrf2 were further examined, and the results showed that most of these genes were upregulated after Nrf2 overexpression, which indicated that Nrf2 exerts antioxidative activity by promoting the transcription of these genes. However, the mRNA levels of GPX1 and catalase (CAT) were not significantly increased, and the mRNA levels of CEBPA, NAPRT and TXNRD1 were unexpectedly decreased dramatically. These results showed that alternative pathways may be responsible for the protective role of Nrf2, which needs further study.

In conclusion, the present study indicated that Nrf2 is critical in the process of RISI. Nrf2 deficiency increases susceptibility to RISI, and forced expression of Nrf2 alleviates RISI after radiation exposure by activating antioxidant enzymes. The present study provides novel evidence for the protective role of Nrf2 in attenuating high-dose radiation-induced RISI.

## Data Availability Statement

The datasets presented in this study can be found in online repositories. The names of the repository/repositories and accession number(s) can be found in the article/**Supplementary Material**.

## Ethics Statement

The animal study was reviewed and approved by Animal Experimentation Ethics Committee at Soochow University (China).

## Author Contributions

JX, CY, WZ and JC conceived and designed the study. JX and CY performed experiments. JX, WM and CY performed data analysis. ZT, YT and WS provided analytic support. ZT and WS provided experimental support. JX, CY, WZ and JC wrote the manuscript with feedback from all authors. All authors contributed to the article and approved the submitted version.

## Funding

This work was supported in part by the National Natural Science Foundation of China grants 81803165 (to JX), 81803039 (to CY), U1967220 (to JC) and 81872552 (to JC); the Natural Science Foundation of Jiangsu Province (Grants No BK20180203 to JX). This study was supported by the Project of State Key Laboratory of Radiation Medicine and Protection, Soochow University (GZK1201809 to JX), Pre-research Program of the 2nd Affiliated Hospital of Soochow University (SDFEYBS1807) and Scientific Research Program for Young Talents of China National Nuclear Corporation (51008).

## Conflict of Interest

The authors declare that the research was conducted in the absence of any commercial or financial relationships that could be construed as a potential conflict of interest.

## Publisher’s Note

All claims expressed in this article are solely those of the authors and do not necessarily represent those of their affiliated organizations, or those of the publisher, the editors and the reviewers. Any product that may be evaluated in this article, or claim that may be made by its manufacturer, is not guaranteed or endorsed by the publisher.

## Supplementary Material

Supplementary Figure 1 The genotypes of the WT (Nrf2^+/+^) and Nrf2-deficient (Nrf2^-/-^) mice were confirmed by PCR analysis.

Supplementary Figure 2 **(A)** Radiation-induced skin injury induced by electron beam irradiation (10 Gy) in WT (Nrf2^+/+^) and Nrf2-deficient (Nrf2^-/-^) mice at 3 days post irradiation. **(B)** Radiation-induced skin injury induced by electron beam irradiation (10 Gy) in WT (Nrf2^+/+^) and Nrf2-deficient (Nrf2^-/-^) mice at 10 days post irradiation.

Supplementary Figure 3 ROS levels in skin fibroblast WS1 cells after IR and Nrf2 overexpression were detected using a ROS-sensitive DCF-DA probe.

Supplementary Figure 4 **(A)** The proliferation of HaCaT cells at 24 hours after irradiation was measured in an EdU incorporation assay by confocal microscopy. **(B)** The proliferation of WS1 cells at 24 hours after irradiation was measured in an EdU incorporation assay by confocal microscopy.

Supplementary Figure 5 **(A)** Representative skin images of the indicated groups at 30 days after 30 Gy irradiation. **(B)** Representative skin images of the indicated groups at 30 days after 45 Gy irradiation.
