# Supplementary figures and images for: NF-E2-Related Factor 2 (Nrf2) Ameliorates Radiation-Induced Skin Injury
Source: Front Oncol. 2021 Aug 23;11:680058. doi: 10.3389/fonc.2021.680058 (PMC8461566; doi:10.3389/fonc.2021.680058)

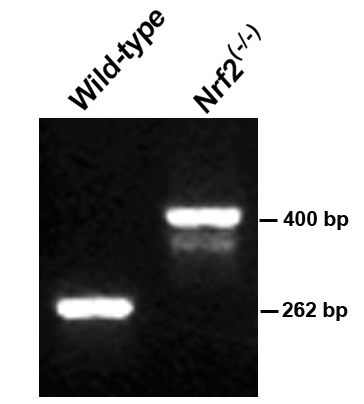

Supplement: Supplementary Figure 1 — The genotypes of the WT (Nrf2+/+) and Nrf2-deficient (Nrf2-/-) mice were confirmed by PCR analysis. [file Image_1.jpeg]

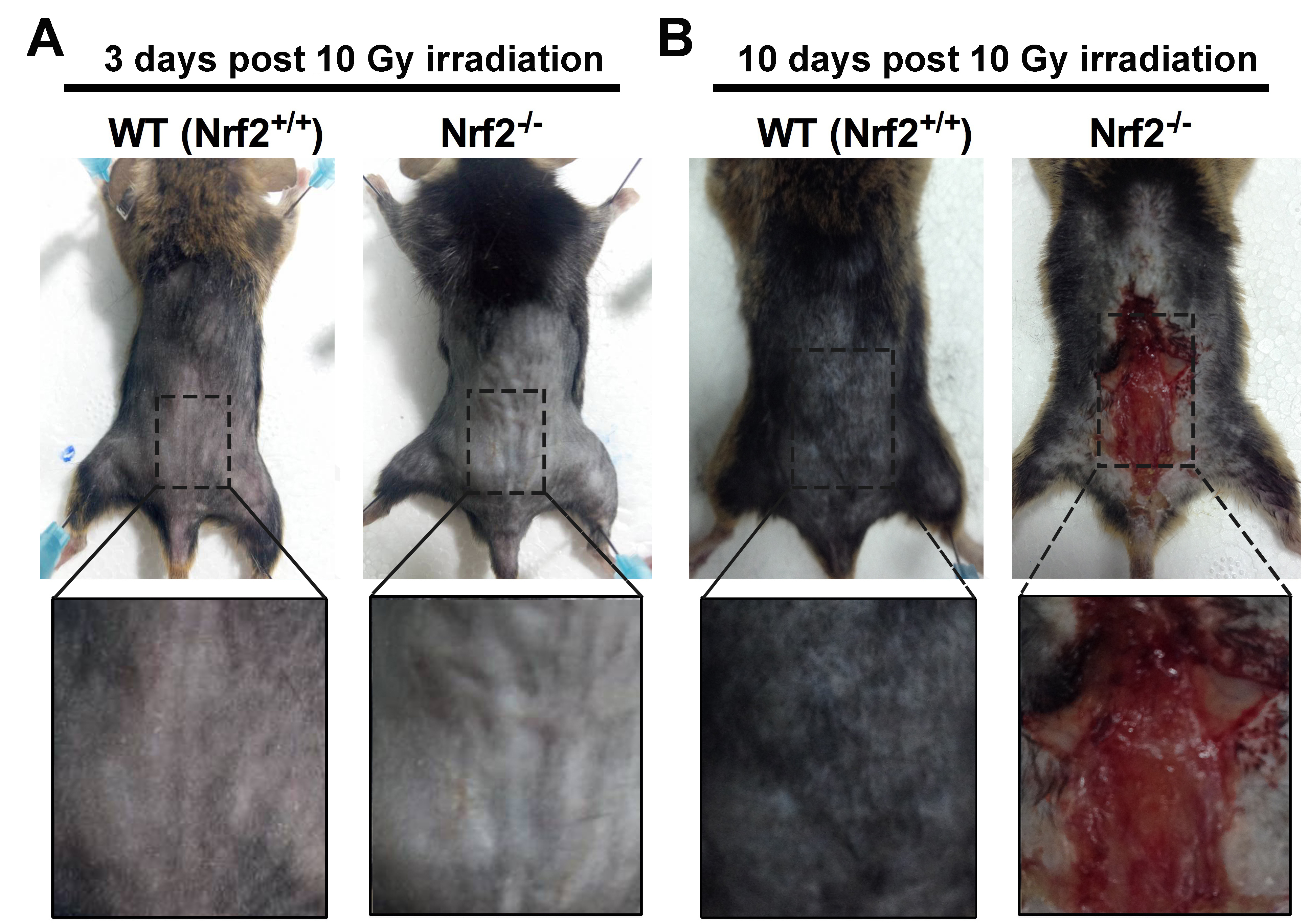

Supplement: Supplementary Figure 2 — (A) Radiation-induced skin injury induced by electron beam irradiation (10 Gy) in WT (Nrf2+/+) and Nrf2-deficient (Nrf2-/-) mice at 3 days post irradiation. (B) Radiation-induced skin injury induced by electron beam irradiation (10 Gy) in WT (Nrf2+/+) and Nrf2-deficient (Nrf2-/-) mice at 10 days post irradiation. [file Image_2.jpeg]

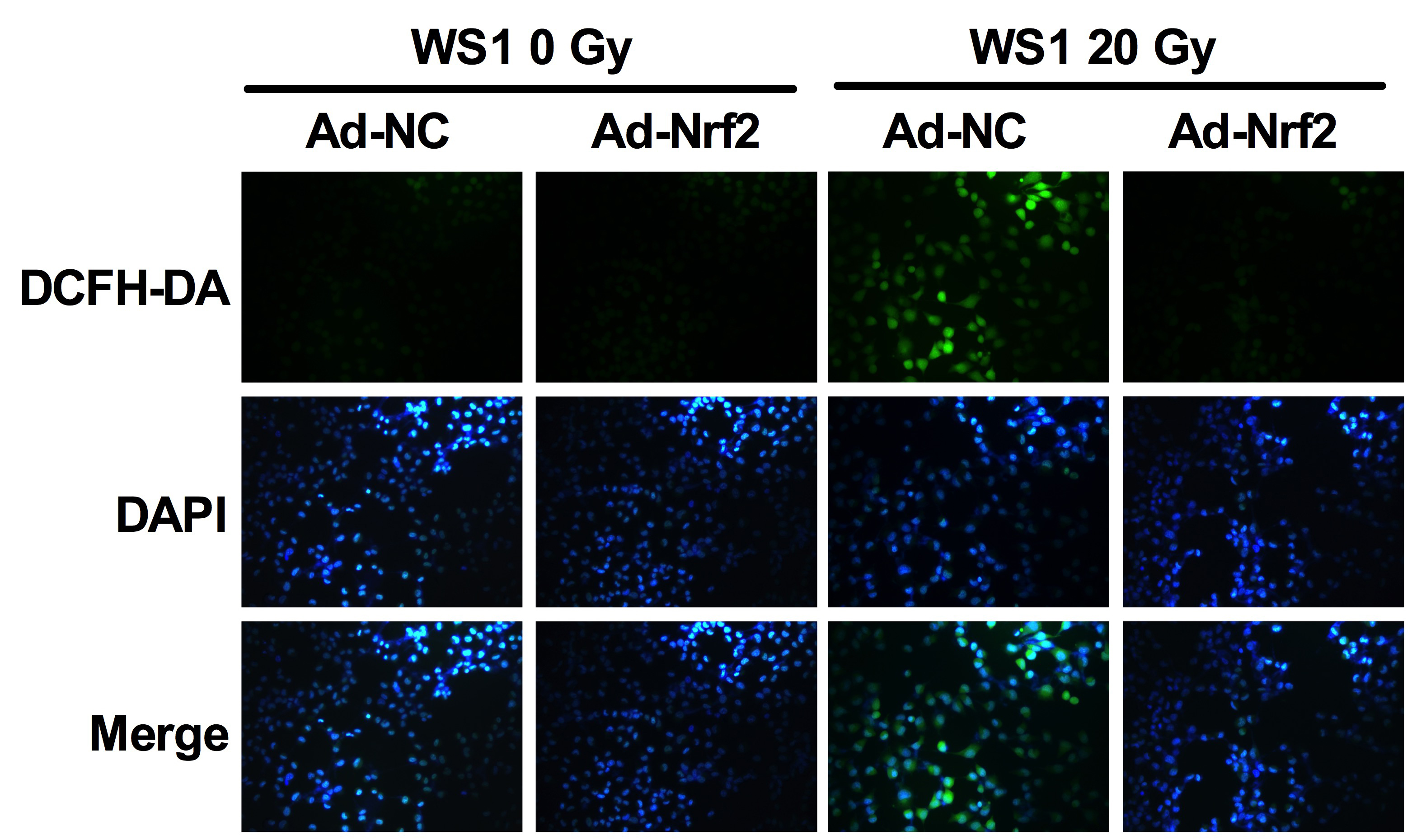

Supplement: Supplementary Figure 3 — ROS levels in skin fibroblast WS1 cells after IR and Nrf2 overexpression were detected using a ROS-sensitive DCF-DA probe. [file Image_3.jpeg]

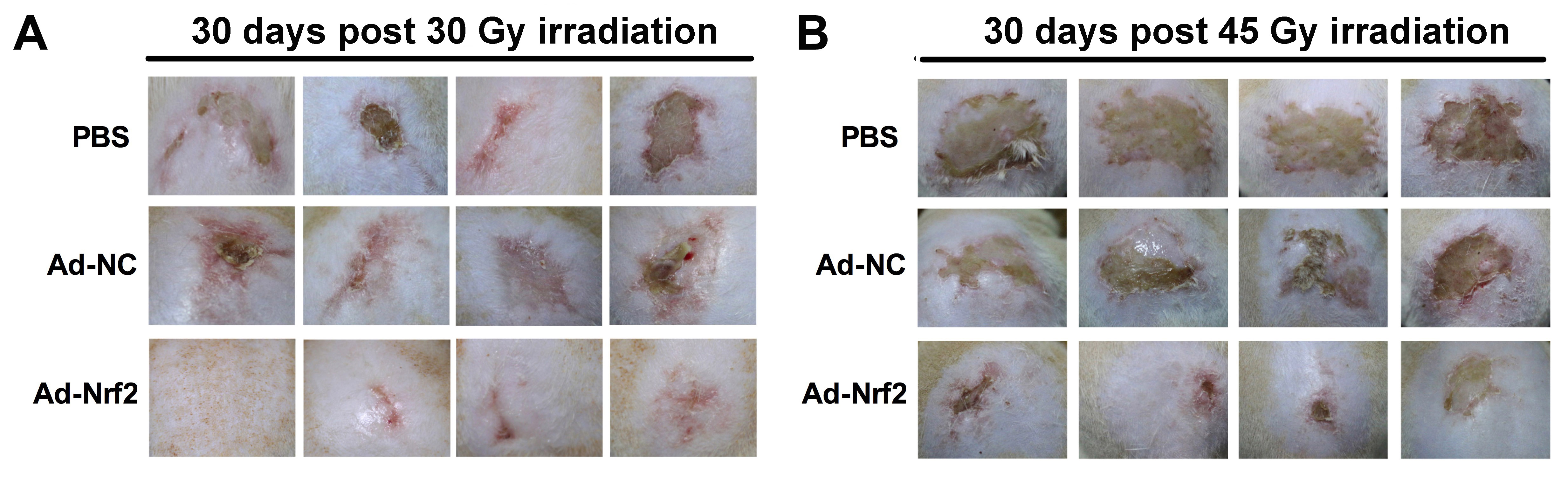

Supplement: Supplementary Figure 5 — (A) Representative skin images of the indicated groups at 30 days after 30 Gy irradiation. (B) Representative skin images of the indicated groups at 30 days after 45 Gy irradiation. [file Image_5.jpeg]
